# Hydroquinone; A Novel Bioactive Compound from Plant-Derived Smoke Can Cue Seed Germination of Lettuce

**DOI:** 10.3389/fchem.2017.00030

**Published:** 2017-05-12

**Authors:** Muhammad Kamran, Abdul L. Khan, Liaqat Ali, Javid Hussain, Muhammad Waqas, Ahmed Al-Harrasi, Qari M. Imran, Yoon-Ha Kim, Sang-Mo Kang, Byung-Wook Yun, In-Jung Lee

**Affiliations:** ^1^School of Applied Biosciences, Kyungpook National UniversityDaegu, South Korea; ^2^UoN Chair of Oman's Medicinal Plants and Marine Natural Products, University of NizwaNizwa, Oman; ^3^Department of Biological Sciences and Chemistry, University of NizwaNizwa, Oman

**Keywords:** plant-derived smoke, seed germination, secondary metabolites, lettuce, *Ginkgo biloba*, column chromatography, hydroquinone

## Abstract

Plant-derived smoke has been known to play an important role in distribution and growth of vegetation. Using a proficiently designed furnace, we extracted smoke from the leaves of four plant viz. *Helianthus annuus*,*Aloe vera*,*Ginkgo biloba*, and *Cymbopogon jwarancusa*. Smoke dilutions obtained from these plants were obtained in different concentrations to identify potential lettuce growth promoting smoke solution. Results revealed that smoke obtained from *G. biloba* significantly enhanced the lettuce seed germination. This solution was then partitioned into ethyl acetate, dichloromethane, *n*-hexane, chloroform and ether fractions. Ethyl acetate fraction was found to be potent to enhance seed germination. This fraction was subjected to column chromatography and spectroscopic techniques to obtain compound **1**. This compound was identified as hydroquinone using 1D and 2D NMR techniques. At low concentrations (5, 10, and 20 ppm), compound **1** enhanced the lettuce seed germination; however, higher concentrations inhibited its growth as compared to control.

## Introduction

Natural fire to vegetation has been identified to enhance seed germination in soil (Light et al., 2004). Plant-derived smoke has been shown to promote seed germination of about 1,200 phylogenetically diverse plant species (Dixon et al., 2009). The first report about role of plant-extracted smoke in the induction of seed germination was published in 1990 (De Lange and Boucher, 1990). Extensive research work has explored various biological roles for plant extracted smoke. Beside germination enhancement of soil seed bank, smoke also increases the seedling vigor even in species where it does not induce seed germination (Brown and van Staden, 1997; Sparg et al., 2005; Stevens et al., 2007; Kamran et al., 2013).

After the initial reports on positive effects of plant-derived smoke, scientists started for hunt of active compounds in smoke solution. Baldwin et al. (1994) Identified 71 compounds in active fractions of smoke by GC-MS and atomic absorption spectrometry. Reports suggested that, the difficulty in isolating the active compounds from plant-derived smoke is due to the presence of large number of compounds (possibly up to several thousands) and partly due to low concentration of the active compound(s) compared to other components (Van Staden et al., 1995). Afterward, Staden et al. (1995) identified about seven different compounds present in both *Passerina vulgaris* and *Themeda triandra* smoke extracts. Among all these, four different compounds were tested on lettuce seed germination assay at concentration ranging from 10^−4^ to 10^−15^ M, but none of them stimulated germination. It was also hypothesized that various gases present in plant-derived smoke such as ethylene, may stimulate flowering (Adriansz et al., 2000). However, other studies claim that ethylene is not the active component of smoke, as ethylene-treated bulbs failed to flower in fire fly (*Cyrtanthus ventricosus*) (Keeley, 1993). Similarly, Keeley and Fotheringham (1997) reported nitrogen oxides present in smoke responsible for stimulation of seed germination. Adriansz et al. (2000) extracted 1,8-cineole as an active germination enhancer. A revolutionary discovery was that of the active compound 3-methyl-2H-furo[2,3-c]pyran-2-one or karrikinolide (KAR_1_) that enhanced germination of a number of species at subnano-molar concentration (Flematti et al., 2004).

The same group explored cyanohydrin glyceronitrile as one of the constituents of plant-derived smoke. Studies have shown that plant-derived smoke and KAR_1_ can influence pre- and post-germination effects in crop seeds such as Z*ea mays* (Sparg et al., 2005), *Allium cepa* (Kulkarni et al., 2010), *Solanum lycopersicum, Abelmoschus esculentus* and beans (van Staden et al., 2006). It also stimulates root growth (Taylor and van Staden, 1998), flowering (Keeley, 1993) and somatic embryogenesis in different crops (Senaratna et al., 1999). However, the pace of new secondary metabolites from smoke is very slow resulting in fewer numbers of bioactive metabolites from smoke of various plant materials.

Although, Karrikins have no exact structural similarity to known phyto-hormones; the “A” ring of the KAR1 molecule is analogous to the “D” ring of strigolactones (Flematti et al., 2004). Studies were conducted to see if Karrikins and strigolactones share similar mechanism of action and it was found that different karrakins (KAR_1_, KAR_2_, KAR_3_, and KAR_4_) showed different responses with KAR2 as the most active one (Flematti et al., 2004). In a recent study (Soós et al., 2010) identified a number of genes that showed differential expression in response to smoke and butenolide including those related to growth and development. In the present study, we aimed to assess the effect of various dilutions of plant-derived smoke on the germination of lettuce seeds. We also aimed to isolate and characterize bioactive metabolites present in the smoke, which can increase the growth of lettuce seeds.

## Materials and methods

### Production of plant derived smoke solution

**Figure 1:**
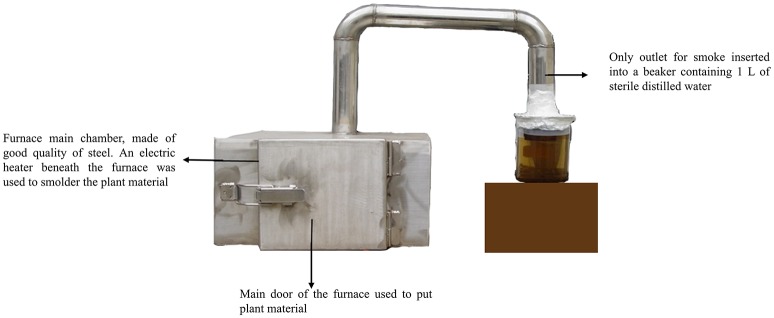
**Furnace design for extraction of plant-derived smoke**. A specialized furnace was designed to extract plant derived smoke. An electric heater was used to produce heat for making smoke of plant material inside the furnace. The smoke was collected in to a glass beaker, containing 1 L DW. The concentrated smoke solution was diluted for further use. The background of the picture was changed for more clarity.

Plant extracted smoke has been used as seed sprouter since 1990s. However, the process of smoke production was in continuous evolution and different methods have been tried so far (Baxter et al., 1994; Thornton et al., 1999; Chumpookam et al., 2012). We designed a furnace that was air tight and the only outlet for smoke was directly immersed in 1 L of water in a beaker (Figure 1). It is important to note that we did not burnt the plant material instead we smoldered it by providing heat through electric heater. Thus, we tried to extract maximum of smoke instead of fire. Plant material of *Helianthus annuus, Aloe vera, Ginkgo biloba*, and *Cymbopogon jwarancusa* were used for smoke solution production.

Each smoke solution was diluted up to 1:500 and was kept at 4 °C for further use. Dilution level was 1:50, 1:100, and 1:500 which was kept at 4 °C for further use. The screening results on lettuce seed germination revealed significant activity of *G. biloba* smoke solution. *G. biloba*, collected from Daegu, Republic of Korea, was dried for 15 days in dark room. Smoke was generated by burning semi-dried *Ginkgo biloba* leaf material (333 g) in the furnace and the smoke was bubbled in one liter of double distilled water (DDW) through a pipe (Kamran et al., 2013). Total 5 l of concentrated smoke solution was produced. The *G. biloba* derived smoke was filtered and kept at 4 °C for further experiments.

### Extraction and isolation

The *Ginkgo biloba* derived smoke solution was screened for the promoting effects on the lettuce seeds. Upon positive germination results of the smoke solution, 100 ml of smoke solution was further extracted with an equal volume of ethyl acetate (EtOAc), dichloromethane (DCM), *n*-hexane, chloroform and ether, three times to obtain extracts. The resulting extracts were diluted to 50, 100, 300, and 500 ppm solutions and were re-examined for bioactivity on lettuce seeds. Among all extracts, ethyl acetate was more active as compared to other extracts and was subjected to chromatography. The rest of the smoke solution (5 l) was extracted with EtOAc three times to obtain a crude gummy extract (6.1321 g). The extract was chromatographed on a 15 g Davisil C18 column (90–130 μm; Alltech, Deerfield, IL, USA) using 50:50 MeOH in water (1.37 g), 30:70 MeOH in water (1.1 g), 10:90 MeOH in water (454 mg), 80:20 MeOH in EtOAc (28 mg), 50:50 MeOH in EtOAc (126 mg), and 30:70 MeOH in EtOAc (89 mg). All six fractions were examined for their germination against the seeds. Fractions 3 (100% MeOH) and 4 (60% MeOH in EtOAc) were bioactive against the germination of lettuce seeds. The sub-fraction obtained at 1:1 water/methanol (1.37 g) was subjected to column chromatography over a silica gel column (50 g, 70–230 mesh, Merck) using 1:9 dichloromethane (DCM):*n*-hexane with gradient in polarity up to pure DCM, then by the gradient of methanol and finally washed with 100% methanol. The chromatographic separations resulted in isolation of compound **1** which was purified at 70% dichloromethane/*n*-hexane system along with some semi-pure fractions using column chromatography. Thin layer chromatography analyses (1:9 MeOH/DCM) confirmed the purity of compound **1**.

The pure compound was identified by the combine use of GC/MS and NMR spectroscopic techniques. The molecular mass and thus the molecular formula was assigned through GC/MS along with ^13^C-NMR data, whereas the individual assignments were carried out by 1D and 2D NMR data.

### Lettuce seed bioassay

Different concentrations of each fraction of EtOAc extract were prepared by dissolving it in autoclaved distilled water (DW). Initial concentration was 1,000 ppm. A glass dish of 27 mm diameter with a lid and a filter paper (27 mm ø, Type Roshi Kaisha, Ltd., Tokyo, Japan) was used in the glass dish. The dilutions were applied on the filter paper and thus allowed to spread over it. Five to seven lettuce seeds were placed on it and the dishes were sealed and packed for incubation for 72 h at room temperature. The control had only DW or 1% DMSO solution. The experiment was repeated three times where each replicate comprised of 20 seeds. For each fraction, mean, SD variance and standard error were calculated to determine inhibition pattern.

### Statistical analysis

All the data were collected from triplicates sequentially repeated three times. The collected data were subjected to DMRT using SAS (Version 9.2, USA) while the graphs were drawn by Graph pad prism (Version 6, USA).

## Results

### Screening of different plant-extracted smoke solutions

**Figure 2:**
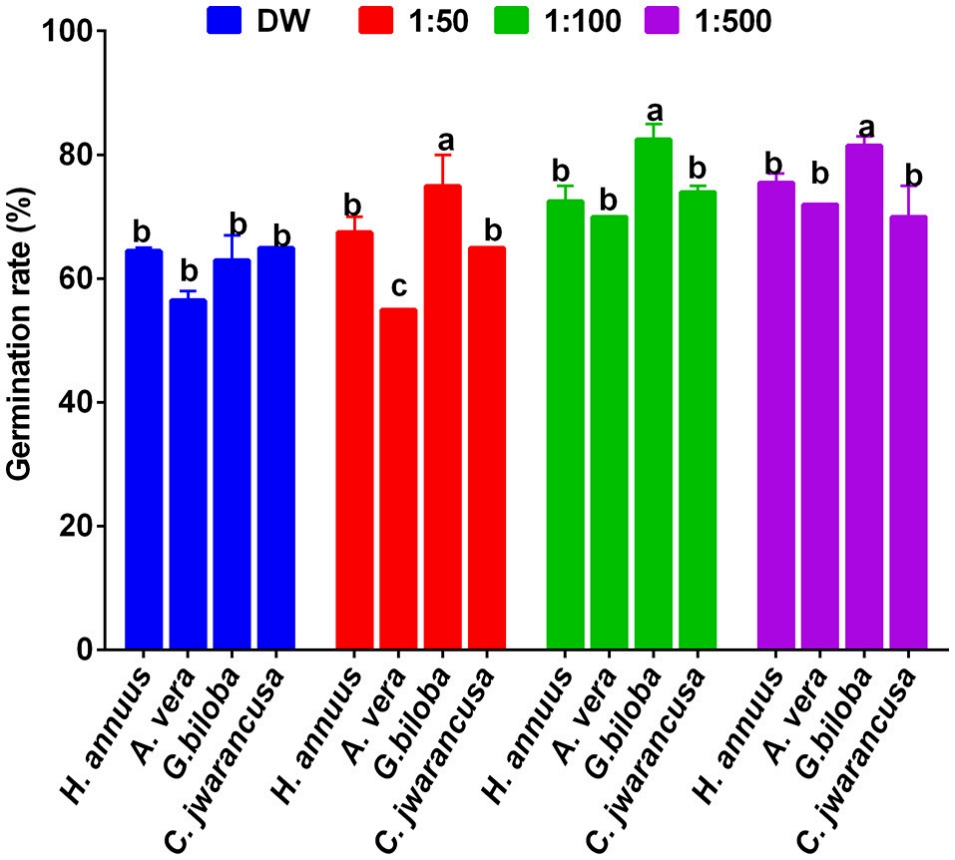
**Effect of various dilutions of smoke solution extracted from ***Helianthus annuus***, ***Aloe vera***, ***Ginkgo biloba***, and ***Cymbopogon jwarancusa*****. The smoke extracts diluted with autoclaved distilled water with 1:50, 1:100 and 1:500; while sole distilled water was used as control. For each set of treatment, the different letters indicates significant differences (*P* < *0.05*) between smoke extracts and control treatments as evaluated by Duncan Multiple Range Test. Error bars refers to SE.

All smoke solutions derived from these four plants along with respective dilutions (1:50, 1:100, and 1:500) were tested on lettuce seed germination and growth. Among smoke solutions, the application of *Ginkgo biloba*-derived smoke showed significantly higher stimulatory effects toward the germination of lettuce seeds (Figure 2). At 1:50, 1:100, and 1:500 smoke solutions, the lettuce seed germination rate was 74, 84, and 85% higher than control, respectively (Figure 2). The smoke solution of *Aloe vera has the least stimulatory effects as* compared to control and other smoke solutions. Based on seed germination results, *Ginkgo biloba* was selected for further chromatographic analysis.

### Identification of bioactive EtOAc fraction

**Figure 3:**
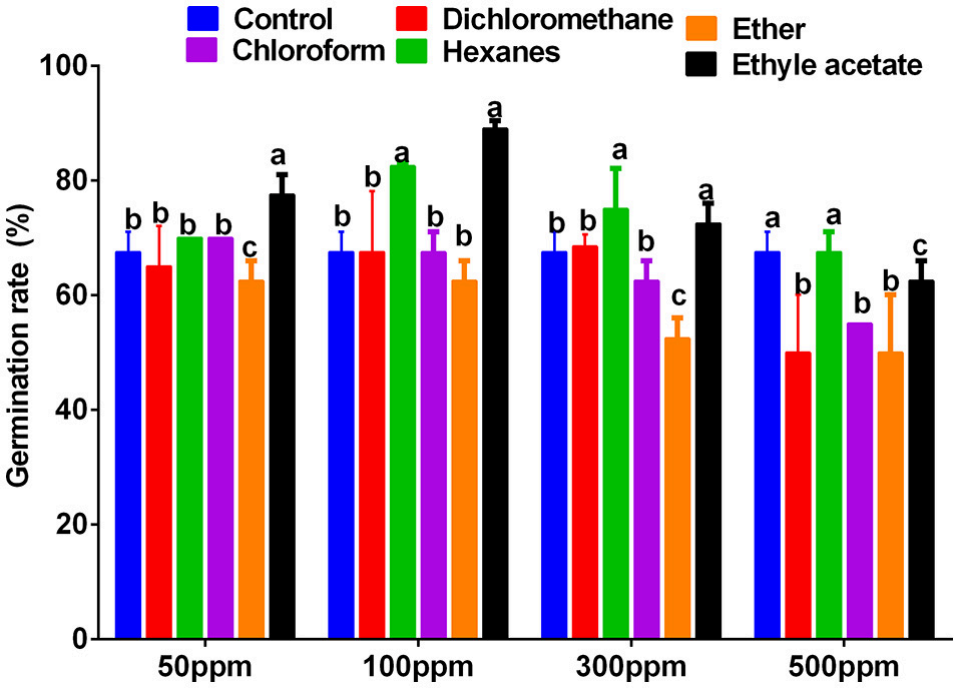
**Effects of ***Ginkgo biloba*** derived smoke extracts (50, 100, 300, and 500 ppm concentration) of ***n***-hexane, ether, dichloromethane, chloroform, and ethyl acetate on lettuce seed germination rate**. Sole distilled water was used only as control against smoke of various concentration extracted in *n*-hexane, ether, dichloromethane, chloroform and ethyl acetate. For each set of treatment, the different letters indicates significant differences (*P* < *0.05*) between smoke extracts and control treatments as evaluated by Duncan Multiple Range Test. Error bars refers to SE.

The 1:100 smoke solution obtained from the plants was partitioned into ethyl acetate, dichloromethane, *n*-hexane, chloroform, and ether fractions. The extracts obtained after solvent-solvent partition (ethyl acetate (EtOAc), dichloromethane, *n*-hexane, chloroform and ether) were again bio-assayed to differentiate bioactive fractions. Various concentrations of 50, 100, 300, and 500 ppm were made to assess the growth promoting effects on the lettuce seeds. The seeds were incubated for 3 days at 25 °C ± 2 in the dark. The control seeds were treated with distilled water. Among all fractions and their concentrations, 50 and 100 ppm of EtOAc fraction showed significantly higher lettuce seed germination as compared to control. However, with increasing concentrations of EtOAc fraction (300 and 500 ppm), the lettuce seed growth decreased as compared to control (Figure 3). The bioactive EtOAc fraction was selected for column chromatography on a C18 column.

### Chromatographic purification of compound 1

The EtOAc fraction was further fractionated into 50:50, 30:70, and 10:90 MeOH in water and 80:20, 50:50, 30:70, and 10:90 MeOH in EtOAc through C18 column chromatography. All six fractions were bio-assayed for their germination against lettuce seeds. Among different concentrations of fraction 1 (50: 50 MeOH in water), 50 ppm was active against the germination of lettuce seeds. The other fractions were either inhibitory or not significantly stimulating to the lettuce seed growth and germination in comparison with control seeds. Fraction 1 was subjected to column chromatography over a silica gel column using 1:9 dichloromethane/*n*-hexane with the gradient increase in polarity up to pure dichloromethane. Methanol gradients were then added, and finally the column was washed with 100% methanol.

### Identification of compound 1 and its bioactivity

**Figure 4:**
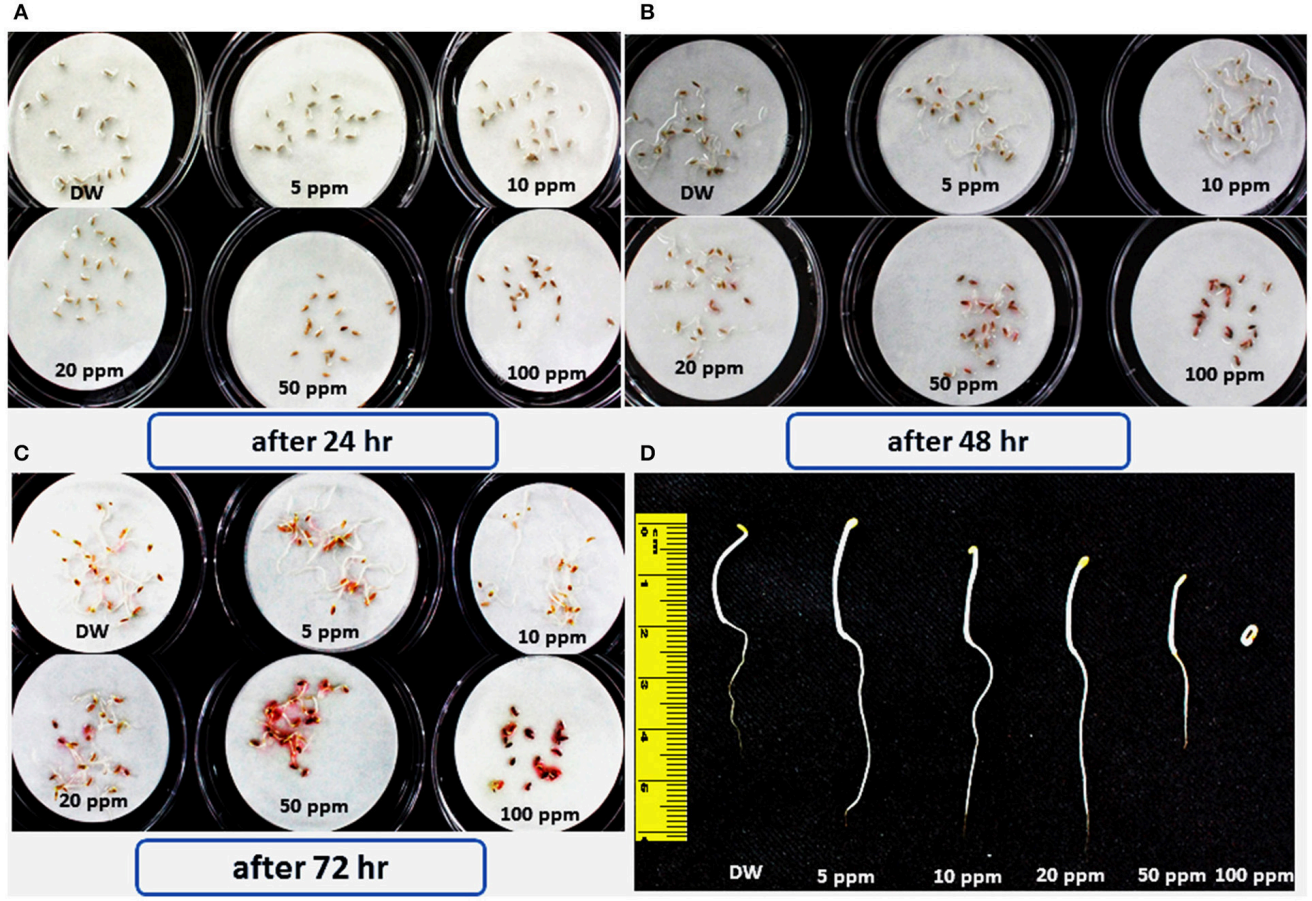
**Effects of various concentrations of bioactive hydroquinone on the germination and growth of lettuce seed; the experiment was repeated three times comprising three replicates having 20 seeds. (A,B,C)** Seed germination response of lettuce seeds in response to 5, 10, 20, 50, and 100 ppm of the bioactive compound 1 (hydroquinone) pictorial presentation shows the data taken after each 24 h. **(D)** Comparative root/shoot length of all tested dilutions of the hydroquinone, compared with control.

These chromatographic separations afforded compound **1** while eluting the column at 7:3 dichloromethane/*n*-hexane solvent system. Compound **1** was bio-assayed for its role in enhancing the lettuce seed germination. The periodic results showed that lower concentrations 5, 10, and 20 ppm have significantly enhanced the seed germination rate as well as significantly increased the shoot and hypocotyl lengths of the lettuce seeds (Figure 4). In contrast, 100 ppm concentration of compound **1** significantly inhibits the growth of lettuce seeds.

### Structure of compound 1 and NMR analysis

**Figure 5:**
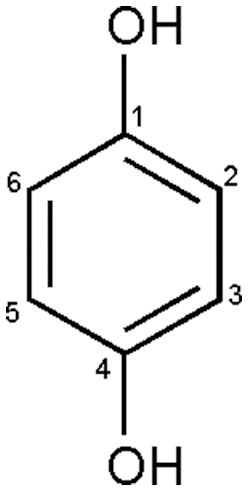
**Structure of compound 1**. Compound **1** was identified as 1,4-dihydroxybenzene also known as hydroquinone by using 1D and 2D NMR spectral data along with the GC/MS analysis and co-TLC with the authentic sample. At low concentrations (5, 10, and 20 ppm) compound **1** enhanced the lettuce seed germination; however, higher concentrations inhibited its growth as compared to control.

Compound **1** was obtained from column chromatography as a light brown crystal. The molecular formula was assigned to be C_6_H_6_O_2_ on the basis of molecular ion at *m/z* 110 in the EI MS and three signals for six carbon atoms in ^13^C NMR spectra of compound **1**. In the ^1^H NMR spectrum, compound **1** exhibited two signals for two protons each in the aromatic region at δ 6.64 (dd, *J* = 6.0, 2.0 Hz, 2 H) and 6.74 (dd, *J* = 6.0. 2.0 Hz, 2 H). The coupling constants and multiplicities of these aromatic protons indicated the *para*-substituted benzene ring in the molecule. The ^13^C NMR spectrum (BB, DEPT) showed three signals, including one oxygenated quaternary carbon signal at δ 146.3 and two sp^2^ hybridized methine signals at δ 116.4 and 120.9. These assignments were further confirmed by relevant HMBC interactions and the structure was finally confirmed as 1,4-dihydroxybenzene, also called hydroquinone (Figure 5). Compound **1** was isolated by repeated column chromatography and the structure was confirmed through MS and NMR spectroscopy.

Compound **1**: Light brown crystal; MP: 171°C; UV (MeOH) λ_max_: 320, 245 nm; IR (KBr): 3595, 2921, 1590, 1465 cm^−1^; ^1^H NMR (300 MHz, CD_3_OD): δ 6.74 (2H, dd, *J* = 6.0, 2.0 Hz, H-2 & H-6); ^13^C NMR (75 MHz, CD_3_OD): δ 146.3 (C-1 & C-4), 120.9 (C-2 & C-6), 116.4 (C-3 & C-5); EI-MS: *m/z* 110 [M]^+^, 92 [M^+^ – H_2_O]^+^; HR-EI-MS: *m/z* 110.1102 [M]^+^ (calcd for C_6_H_6_O_2_, 110.1106).

**Figure 6:**
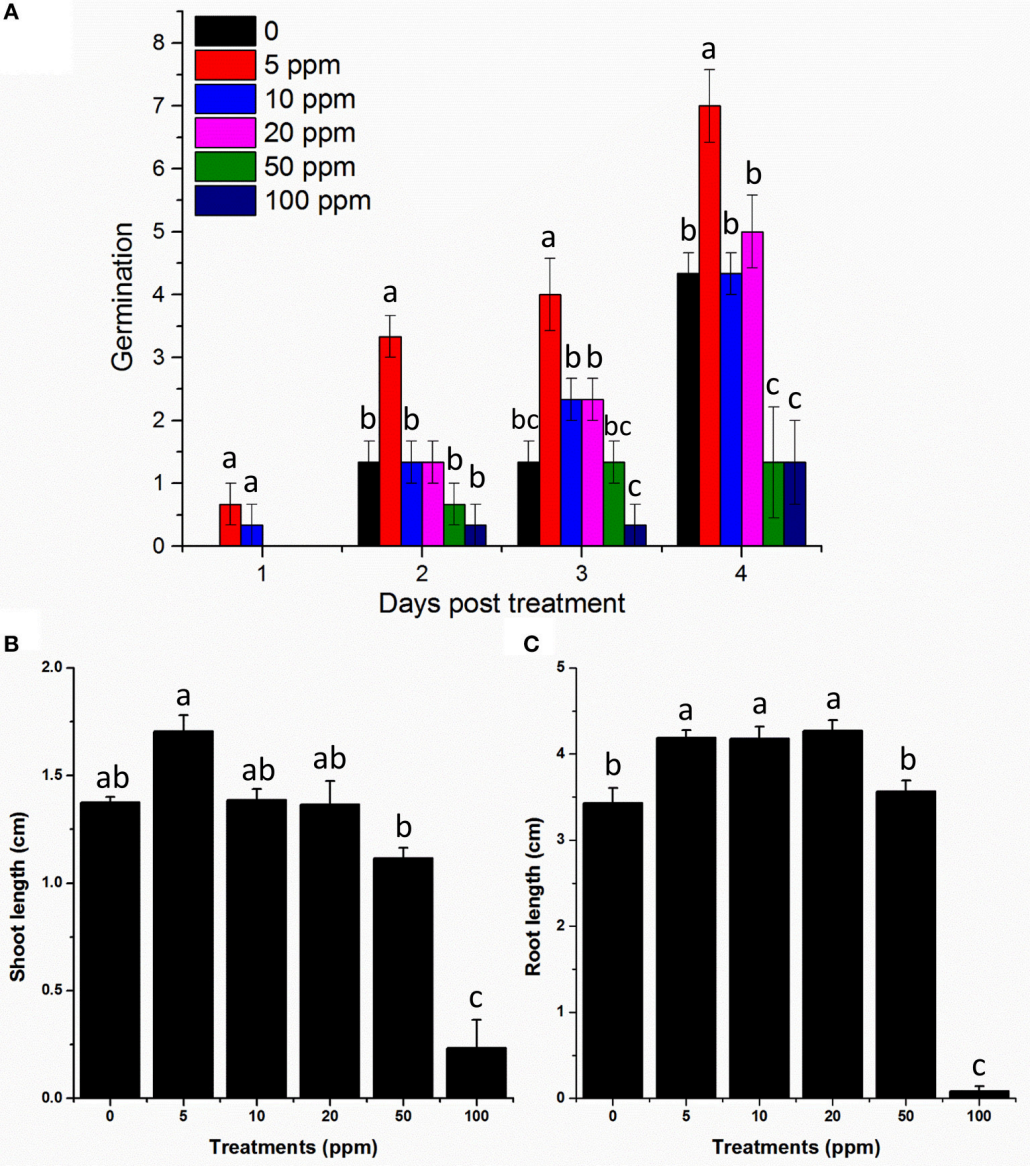
**Germination and growth assay of commercially available hydroquinone on lettuce seeds. (A)** Germination rate of lettuce under the indicated hydroquinone treatments. **(B)** Shoot and **(C)** root length of lettuce in response to the indicated treatments. All the treatments are in ppm. 0 mean control plants treated with distilled water only. The error bars represents ± SE and different letters indicates significant differences (*P* < *0.05*).

To verify the structure and bioactivity, lettuce seeds were exposed to commercially available Hydroquinone (Sigma-Aldrich) and compared with the natural product isolated from plant-derived smoke. Our results suggested higher germination rate at 5 ppm treatment compared to control until 4 days (Figure 6). Similarly, shoot and root length was also examined and we found that 5 ppm also enhanced lettuce shoot length compared to control. The minimum shoot length was found for 100 ppm treatment. Furthermore, in case of root length, 5, 10, and 50 ppm showed higher root length compared to control (Figure 6). However, the highest concentration i.e., 100 ppm again showed significant reduction in root length compared to all other treatments (Figure 6). These results suggest a similar effect to those of our isolated product thus supporting the characterization and bioactivity results, and hence the structure is confirmed.

## Discussion

In the present study, we isolated, identified and characterized hydroquinone from the plant-derived smoke of *Ginkgo biloba* through a bioassay-guided approach. Previously, the same compound was identified from many other sources, including animals (*Castor Canadensis*), plants (*Ecdysanthera rosea*) (Zhu et al., 2010) and microbes (*Agaricus hondensis*), however, its isolation from the smoke is reported for the first time here. Although it has been known to play essential role as an oxidizing and reducing agent, and it is frequently used in cosmetic industries for skin whitening, its role in agriculture as a seed germination enhancer is rarely known. Previously it was reported (Elwakil, 2003) that the application of 20 mM hydroquinone not only increases seed germination of peanut but also inhibits the growth of seed-born fungi. Another study showed that treatment of 0.25 mM hydroquinone increased the stomatal resistance, lowered the transpiration, and reduced the growth of soybean seedlings (Barkosky et al., 1999). However, hydroquinone with concentration of 0.1 mM resulted in inhibiting the growth of leafy spurge. In relation to the present study, Li et al. (2009) identified hydroquinone from *Podophyllum hexandrum*, which was found to enhance wheat seedling growth (Li et al., 2009). A similar pattern of effect was also observed for *P. vulgaris*, when 0.25 mM of hydroquinone inhibited the chlorophyll fluorescence and membrane potential (Keller et al., 2008). The literature revealed that hydroquinone is suppressive to plants but at the same time, it ameliorates growth and germination of seeds. Our results also showed a similar pattern of increased lettuce seed growth at lower concentrations and inhibition on higher concentrations. The present results of our experiments were in agreement with the previously reported results, which suggested that the lower concentration of hydroquinone application can cue seed germination and seedling growth.

Increased stimulation of weed seeds in agricultural fields can help reduce their numbers in soil seed banks (Dyer, 1995). This could be a more environmentally friendly weed management strategy (Stevens et al., 2007). Plant derived smoke has been shown to promote seed germination of a broad spectrum of weed species (Adkins and Peters, 2001; Baker et al., 2005; Daws et al., 2007; Stevens et al., 2007) and crop species (Drewes et al., 1995; Thomas and Van Staden, 1995; van Staden et al., 2006). Therefore, targeting soil seed banks for uniform and quicker weed seed germination and seedling establishment using plant-derived smoke could be an effective alternative to weed management (Figure 1). In the present study, plant-derived smoke was extracted into solution and evaluated for possible effects against barnyard grass seed germination. Upon confirmation of its effects, its mechanism of action was investigated.

In conclusion, the present results showed that plant-derived smoke can influence seed germination and growth. Using a bioassay-guided approach, hydroquinone (compound **1**) was isolated, identified and characterized from plant-derived smoke extract through advanced chromatographic and spectroscopic methods. Hydroquinone was observed to increase seed germination and growth on lower concentrations; however, at higher concentrations it is inhibitory to the seed germination and growth. Among some of the already reported seed cueing substances from smoke, it is the first one which might play an essential role in fire and agricultural ecology. Further experiments related to different crops and effects on their growth will unveil the broader application of this eco-friendly constituent.

## Author contributions

IL and BY supervised the research. MK, AK, IL, and BY conceived the project. MK, QMI, MW, LA, and AK planned, designed and performed experiments. AA-H, YK, JH, and SK analyzed data. MK, QMI and AK drafted sections.

### Conflict of interest statement

The authors declare that the research was conducted in the absence of any commercial or financial relationships that could be construed as a potential conflict of interest.
